# Supplementary material for: Early Detection of Therapeutic Benefit from PD-1/PD-L1 Blockade in Advanced Lung Cancer by Monitoring Cachexia-Related Circulating Cytokines
Source: Cancers (Basel). 2023 Feb 11;15(4):1170. doi: 10.3390/cancers15041170 (PMC9954513; doi:10.3390/cancers15041170)
Supplement: Supplementary file 1 [file cancers-15-01170-s001.zip › cancers-2178675-supplementary.pdf]

cancers

MDPI

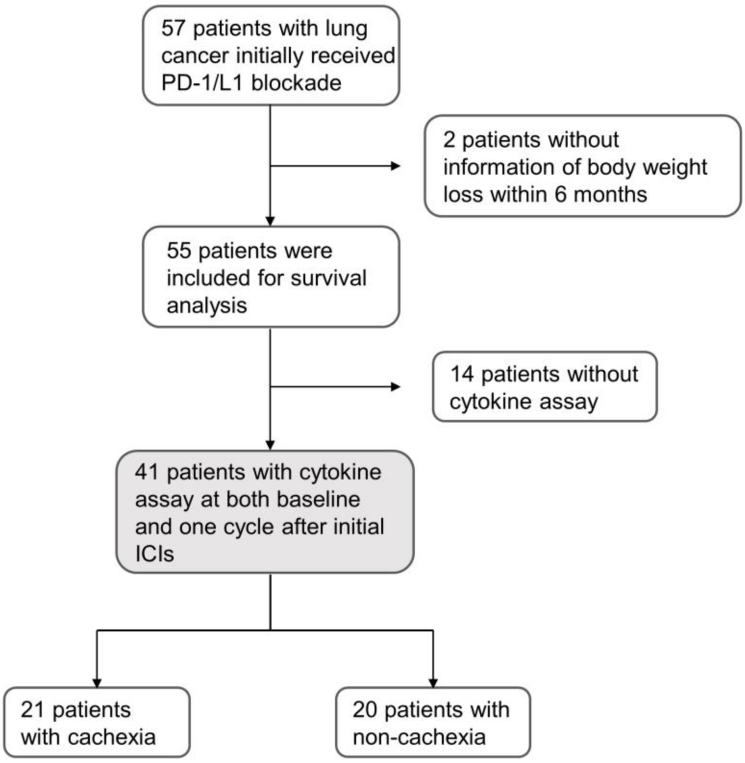

Figure S1. Study flow diagram.

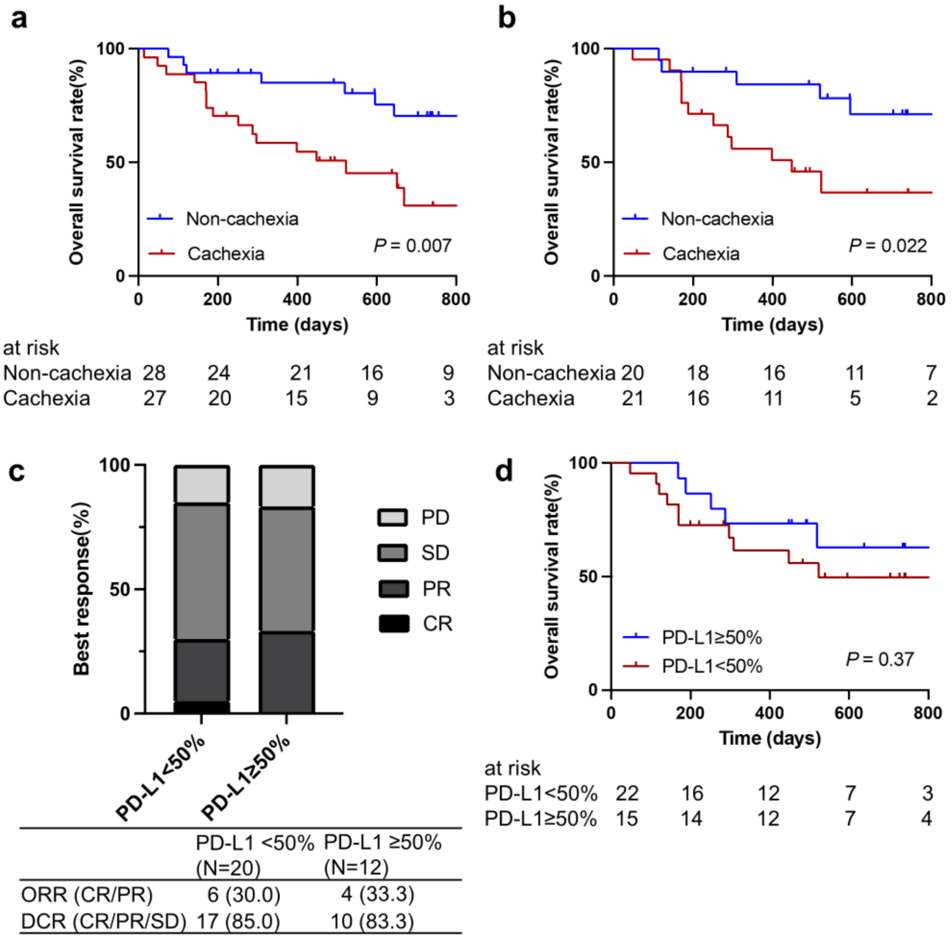

Figure S2. The impact of cancer cachexia and PD-L1 expression on therapeutic outcomes (a) Overall survival in patients with cachexia (n=27) versus those without cachexia (n=28) in the cohort of 55 patients. (b) Overall survival between cachectic (n=21) and non-cachectic (n=20) patients in 41 patients with a cytokine assay analysis. (c) Therapeutic responses to PD-1/PD-L1 blockades according to PD-L1 expression in 41 patients with a cytokine assay analysis. (d) Overall survival in patients with high PD-L1 (≥50%) versus those with low PD-L1 (<50%).

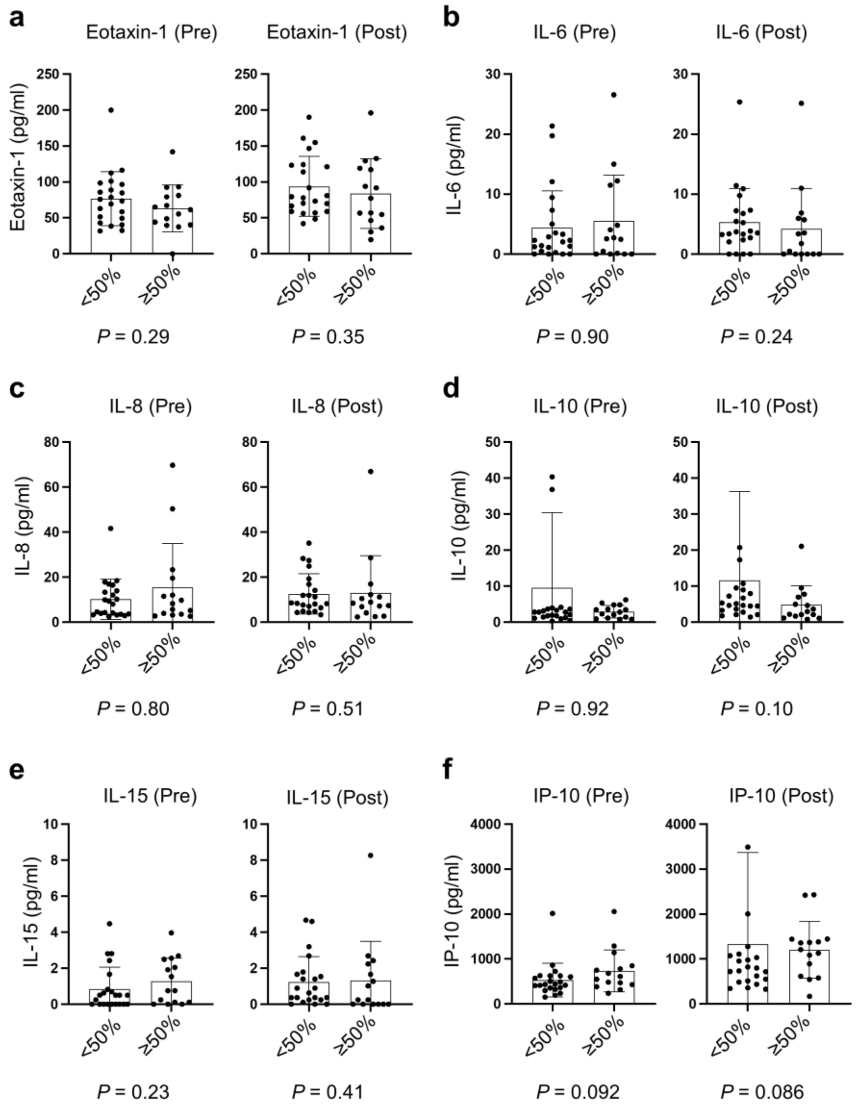

Figure S3. Distribution of cachexia-related cytokines in PD-L1 < 50% versus PD-L1 ≥ 50% at baseline and during treatment with PD-1/PD-L1 blockade.
